# Case Report: Familial Cold Autoinflammatory Syndrome With Double Variant in *NLRP12* and *SETD1A*

**DOI:** 10.1155/carm/2545876

**Published:** 2026-05-18

**Authors:** José Eduardo Ruíz-Santana, Oscar Aquino-Arango, María Fernanda Alvarado-Fernández, María del Carmen Chima-Galán, Fernando Lozano-Patiño, María Eugenia Vargas-Camaño, María Isabel Castrejón-Vázquez

**Affiliations:** ^1^ Department of Clinical Immunology and Allergy, National Medical Center “20 de Noviembre”, ISSSTE, Mexico City, Mexico, issste.gob.mx; ^2^ Faculty of Medicine, Universidad Nacional Autónoma de México (UNAM), Mexico City, Mexico, unam.mx; ^3^ Department of Genomic Medicine, National Medical Center “20 de Noviembre”, ISSSTE, Mexico City, Mexico, issste.gob.mx

## Abstract

The inflammasome is a protein complex involved in the activation of inflammatory responses through the production of proinflammatory cytokines and pyroptosis. *NLRP12* is a NOD‐like receptor that regulates inflammation and inflammasome signaling. Mutations in the *NLRP12* gene can lead to familial cold autoinflammatory syndrome type 2 (FCAS2), an autosomal dominant disorder characterized by episodes of fever, urticaria, arthritis, and symptoms triggered by cold exposure. This work presents the case of a pediatric patient with a history of recurrent infections, allergies, and epileptic seizures associated with immunoglobulin administration. Exome sequencing identified two variants: one in the *SETD1A* gene, associated with neurodevelopmental disorders, and another in *NLRP12*, previously reported as benign. However, due to the clinical presentation compatible with FCAS2 and signs of immunodeficiency, it is suggested that the *NLRP12* variant might have an underestimated pathogenic role. This case highlights the need for further studies to better understand the clinical variability of autoinflammatory diseases and their relationship with genetic variants, as well as the importance of developing targeted treatments.

## 1. Introduction

Inflammation is a physiological response to infection and tissue damage, essential for eliminating pathogens and promoting tissue repair, and can be triggered by the activation of pattern recognition receptors (PRRs) expressed by cells of the innate and adaptive immune systems. These receptors detect highly conserved microbial components, known as pathogen‐associated molecular patterns (PAMPs), or host‐derived molecules released during stress or tissue injury, referred to as damage‐associated molecular patterns (DAMPs) [1, 2].

Among the PRRs are the NLRs, such as NLRP1, NLRP2, NLRC3, NLRP4, NLRP6, NLRP7, NLRP10, NLRP12, and NLRX1. They perform various functions, such as acting as crucial components of inflammasomes [1, 3, 4]. Inflammasomes are cytosolic protein complexes that detect nonspecific signals through NLRs. They consist of a sensor molecule (NLRs), an adaptor molecule (ASC), and an effector molecule (caspase‐1). Activation of the sensors triggers the recruitment of ASC, leading to the activation of procaspase‐1, which results in the cleavage and secretion of proinflammatory cytokines such as pro‐IL‐1β and pro‐IL‐18 into IL‐1β and IL‐18, respectively, as well as pyroptosis [2].

**FIGURE 1:**
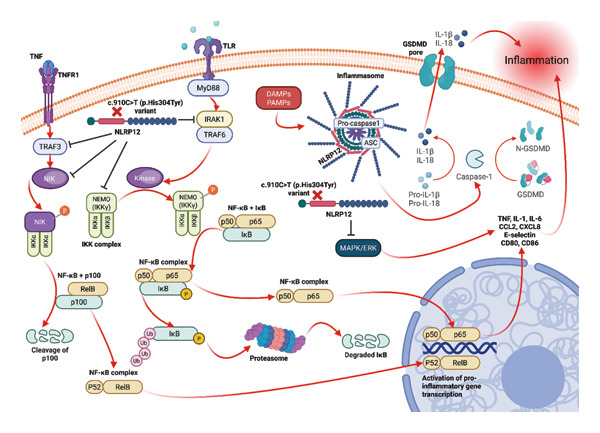
Schematic representation of the *NLRP12* protein structure and its role in inflammasome‐mediated inflammatory pathways. *NLRP12* (NOD‐like receptor family pyrin domain‐containing 12) encodes a member of the NOD‐like receptor (NLR) family, primarily expressed in immune cells and involved in the regulation of innate immune responses. Upon activation, *NLRP12* can participate in inflammasome assembly, contributing to caspase activation and subsequent processing and secretion of interleukin‐1β (IL‐1β). The location of the p.His304Tyr variant identified in our patient is illustrated within the NACHT domain, a region critical for nucleotide binding and oligomerization. Although classified as benign in current databases, alterations in this domain have been associated with dysregulated inflammatory signaling in previous reports. This schematic is intended to provide biological context for the potential mechanisms by which variants in NLRP12 may be associated with autoinflammatory manifestations observed in the present case. Created in BioRender. Ruíz Santana, J. E. (2026) https://BioRender.com/gxjcfys.

The *NLRP12* gene, located on the long arm of chromosome 19 (19q13.42), encodes the *NALP12* receptor (also known as RNO, PYPAF7, or Monarch‐1), which is a member of the cytoplasmic protein family CATERPILLER [5–7]. NLRP12 is involved in the regulation of inflammation and innate immune responses by contributing to the activation, processing, and secretion of caspases and the overproduction of IL‐1β. It is expressed in myeloid lineage cells and is downregulated in response to pathogens and inflammatory cytokines. *NLRP12* antagonizes canonical and noncanonical NF‐κB signaling, alters cell migration, suppresses proinflammatory cytokine production, and regulates MHC‐I gene expression [1, 5, 7–9]. (Figure 1).

Autoinflammatory syndromes (AIS) are a group of rare monogenic diseases characterized by excessive production of proinflammatory cytokines due to abnormal activation within the innate immune system [7, 10–12].

Patients with gain‐of‐function (GOF) mutations in the *NLRP12* gene develop a rare autosomal dominant genetic disorder, familial cold autoinflammatory syndrome type 2 (FCAS2, OMIM #611762), an AIS primarily triggered by cold exposure [3, 6, 9, 10, 12].

Mutations in the *NLRP12* gene lead to accelerated IL‐1β secretion in monocytes due to increased NF‐κB activity. Additionally, hyperresponsiveness to stress, alterations in redox reactions resulting in higher levels of reactive oxygen species (ROS), and antioxidants have been observed [6, 8, 9, 13].

The clinical characteristics of FCAS2 include paroxysmal periodic fever, myalgia, arthralgia, headache, arthritis, lymphadenopathy, abdominal pain likely associated with peritonitis, chronic diarrhea, chest pain associated with pleuritis, skin manifestations (such as urticaria), susceptibility to infections, and an increased long‐term risk of developing amyloidosis, as well as systemic inflammation without high titers of autoantibodies or elevated levels of antigen‐specific T lymphocytes. Other less common symptoms include splenomegaly, sensorineural hearing loss, and reduced visual acuity. A marked increase in acute‐phase reactants (CRP and ESR) has also been described [3, 7, 12].

Most cases appear in childhood; however, some patients develop symptoms only in adulthood. Clinical manifestations may primarily occur during winter and can be exacerbated by cold exposure [6, 9, 13].

The diagnosis is based on recognizing recurrent noninfectious fever, especially with a pattern of clinical manifestations (such as urticaria) triggered by cold exposure. Suspicion increases when there is a positive family history [12].

## 2. Case Presentation

A 5‐year‐old female preschooler, originally from the United States and currently residing in Mexico, was evaluated. Her father has a history of atopic dermatitis and food allergies, and her sister has food allergies. There was no history of consanguinity or family history of seizures or cold‐induced rash. She was the product of her mother’s third pregnancy at 35 years of age and her father at 37 years of age. The pregnancy was complicated by a threatened miscarriage at 12 weeks of gestation. She was delivered by cesarean section at 39 weeks without complications.

The umbilical cord detached at 2 weeks of age. She was exclusively breastfed for 6 months, followed by the introduction of complementary feeding without complications. Beginning at 8 months of age, she experienced recurrent upper respiratory and gastrointestinal infections, approximately three episodes per month for 1 year, requiring hospitalization on one occasion.

At 2 years of age, she developed a herpes virus infection treated with topical acyclovir for 7 days, with clinical improvement. One month later, she presented with cutaneous larva migrans on the right foot and, 4 months afterward, helminth expulsion in the perianal region. Both parasitic infections were treated with ivermectin, with resolution.

Laboratory evaluation revealed hypogammaglobulinemia: IgA 20.8 mg/dL (reference range for age: 14–123 mg/dL [14]), IgG 393.7 mg/dL (reference range: 424–1051 mg/dL [14]), and IgG3 8.9 mg/dL (reference range: 9–63 mg/dL [15]).

**FIGURE 2:**
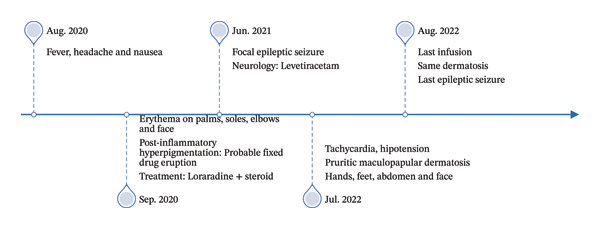
Timeline showing the clinical progression of the patient over time.

Monthly intravenous immunoglobulin (IVIG) replacement therapy was initiated. During the first infusion, the patient developed a headache followed by a generalized tonic–clonic seizure lasting approximately 60 s. In the postictal period, she presented with fever and a generalized erythematous, pruritic maculopapular rash involving the face, trunk, and extremities, which improved with antihistamines and systemic corticosteroids. Levetiracetam was initiated (Figure 2).

IVIG therapy continued monthly with premedication for 2 years. During this period, she experienced similar dermatologic reactions in most infusions and seizures on two additional occasions. Upon further questioning, the patient’s mother reported that a similar rash occurred after bathing. During the last two IVIG infusions, the patient also developed hypotension associated with the rash, prompting referral to our service with a suspected adverse reaction to immunoglobulin.

Physical examination revealed normocephaly with subtle dysmorphic features, including frontal bossing, arched eyebrows, horizontal palpebral fissures, low and broad nasal bridge, bulbous nasal tip, bilaterally flattened zygomatic arches, short columella, and dental diastema of the upper and lower incisors. No lymphadenopathy or organomegaly was detected. Cardiopulmonary, abdominal, musculoskeletal, and neurologic examinations were otherwise unremarkable. Skin was adequately hydrated.

IVIG was switched to subcutaneous immunoglobulin (SCIG), resulting in resolution of the rash. Laboratory tests (Table 1) showed IgG levels within the normal range, likely reflecting ongoing immunoglobulin therapy, which was not discontinued. IgM levels were decreased by more than two determinations. B lymphocytes were initially reduced, with subsequent normalization, raising suspicion for common variable immunodeficiency.

**TABLE 1:** Laboratory results of the patient over time.

Laboratory/date	03.11.2022	17.01.2023	24.03.2023	29.05.2023	11.08.2023	13.10.2023	Reference range for age
IgG (mg/dL)	725	918	727	1050	950	1090	463–1236 [14]
IgM (mg/dL)	44.7	**36.8** ^**∗**^	**39.4** ^**∗**^	50.7	77.8	49.0	43–196 [14]
IgA (mg/dL)	48.3	32.0	43.8	54.7	64.6	51.2	25–154 [14]
IgE (UI/mL)	94.9	61.8	59.9	85.5	301	156	1.07–68.9 [16]
CD3^+^ T Lymphocytes (cel/μL)	2161	1211	2174		2981	2370	1400–3700 [17]
CD4^+^ T Lymphocytes (cel/μL)	1328	789	1347		1967	1394	700–2200 [17]
CD8^+^ T Lymphocytes (cel/μL)	698	**353** ^**∗**^	671		858	806	490–1300 [17]
CD4^+^/CD8^+^ Ratio	1.90	2.23	2.0		2.29	1.73	
CD16^+^CD56^+^ Cells (cel/μL)	157	**82** ^**∗**^	267	308	133	313	130–720 [17]
CD19^+^B Lymphocytes (cel/μL)	**389** ^**∗**^	**287** ^**∗**^	**379** ^**∗**^	397	509	459	390–1400 [17]

*Note:* The bold values marked with asterisks (^∗^) do not indicate statistical significance. They were used to highlight laboratory values outside the age‐adjusted reference ranges (decreased). This notation is intended for clinical interpretation and to facilitate identification of abnormal findings.

Despite therapy, the patient continued to experience recurrent upper respiratory and gastrointestinal infections, new parasitic infections, and episodes of headache and fever following SCIG administration.

Given the suspicion of an inborn error of immunity characterized by predominant antibody deficiency and autoinflammatory features, whole exome sequencing (WES) was performed. Copy number variation analysis or molecular karyotyping was not performed. Two variants were identified: a pathogenic variant in *SETD1A* (c.4582‐2_4582‐1del) and a variant in NLRP12 (c.910C > *T*; p.His304Tyr).

## 3. Discussion

*NLRP12* is a complex protein with multiple roles in inflammation, cancer, infections, and autoinflammatory disorders. By interacting with other molecules, it can form a cytosolic protein complex known as the inflammasome, which, when activated, leads to the production of proinflammatory cytokines (pro‐IL‐1β and pro‐IL‐18) and pyroptosis. Inflammasome‐mediated inflammation is a critical mechanism that, if altered, can lead to the development of AIS [1, 5, 7–12].

Pathogenic variants in *NLRP12* can cause FCAS2, a rare autosomal dominant systemic autoinflammatory disorder characterized by urticaria, arthritis, fever, and leukocytosis (sometimes triggered by cold exposure) [3, 6, 9, 10, 12].

Most known variants in *NLRP12* are missense mutations, often located in the NACHT region, which can affect its immunomodulatory function. These are primarily found in exon 3, with the most frequently reported variant being c.1206C > G, p.(Phe402Leu) [p.F402L.13]. However, these variants may exhibit low penetrance (possibly due to haploinsufficiency) and may require additional factors for clinical manifestation of the disease [3, 8, 9, 11].

Some patients with loss‐of‐function mutations in *NLRP12* may exhibit reduced production of proinflammatory cytokines (IL‐1β, IL‐6, TNF‐α) in response to specific stimuli. Counter‐regulatory cytokines (IL‐10, sTNFRII, and TGF‐β) are usually unaffected. Additionally, certain *NLRP12* variants may be associated with low levels of IgG, IgA, and/or IgM [5, 7, 10, 18].

Patients with compatible symptoms, family history, and response to treatment should undergo genetic testing, such as next‐generation sequencing (NGS), to confirm the presence of pathogenic or likely pathogenic variants in the *NLRP12* gene [8, 9]. However, despite the above, to date, there are no validated classifications or diagnostic criteria to aid in the diagnosis of this disease. Although cutaneous manifestations are the most common, it can cause other manifestations such as abdominal pain, diarrhea, hepatosplenomegaly, lymphadenopathy, aphthous stomatitis, and headache, among many others, sometimes accompanied by other comorbidities [19].

*SETD1A* encodes a SET domain–containing protein that forms part of a histone methyltransferase enzymatic complex responsible for mediating trimethylation of histone H3 at lysine 4 (H3K4me3). Through this epigenetic regulatory function, *SETD1A* plays a critical role in cell cycle control and genome stability, and has been implicated in the regulation of neuronal progenitor cell proliferation [20–22].

Pathogenic variants in *SETD1A* have been associated with global developmental delay, intellectual disability, subtle facial dysmorphisms, behavioral and psychiatric disorders (including schizophrenia), and, in some cases, tumor susceptibility [20, 22–26].

The variant identified in our patient is a pathogenic *SETD1A* mutation associated with an autosomal dominant neurodevelopmental disorder characterized by speech impairment, dysmorphic facial features, and epilepsy [20, 23–26]. In this context, the early‐onset, difficult‐to‐control epilepsy and subtle facial dysmorphisms observed in our patient can be reasonably attributed to the known clinical spectrum of *SETD1A*‐related disease.

Importantly, these neurodevelopmental manifestations are distinct from the autoinflammatory features described in association with *NLRP12* variants. While the coexistence of pathogenic variants in two different genes may contribute to a complex phenotype, the current evidence does not allow determination of a direct interaction between *SETD1A* and *NLRP12*. Therefore, in this case, the neurodevelopmental features are most consistent with the pathogenic *SETD1A* variant, whereas the autoinflammatory and immunological findings may be associated with the *NLRP12* variant, acknowledging the limitations previously discussed.

Although the *NLRP12* variant identified in our patient is classified as benign in several databases, cases have been reported in China, Turkey, Russia, Brazil, Italy, and Ecuador describing patients carrying the same variant who presented clinical manifestations consistent with autoinflammatory disease and FCAS [7, 19, 27–32]. Additionally, an in vitro functional study of the NLRP12 protein in a patient carrying the same variant demonstrated increased IL‐1β secretion, suggesting potential impairment of protein function [28]. These observations suggest a possible association between this variant and autoinflammatory phenotypes; however, current evidence remains insufficient to establish a definitive causal relationship.

The clinical manifestations in our patient appear milder compared to those typically observed in individuals carrying clearly pathogenic *NLRP12* variants. It has been proposed that low‐penetrance variants may be associated with attenuated or variable phenotypes in some patients with autoinflammatory manifestations. Additionally, interfamilial clinical heterogeneity may be more pronounced among carriers of such variants. Therefore, family segregation studies and functional assays are essential to clarify their clinical significance [19]. Unfortunately, these analyses were not available for our patient.

The correlation between genotype and phenotype is complex and, to date, remains not fully understood. Additionally, the coexistence of multiple variants in different genes may contribute to the complex clinical manifestations [6, 10].

Although FCAS2 is a rare AIS, further studies are needed to better define its diagnostic framework. Although the patient’s *NLRP12* variant is classified as benign, her clinical presentation is compatible with FCAS2. Notably, episodes of rash following bathing and IVIG infusion may be temporally associated with decreased body temperature.

Additionally, there is a case report in the United Kingdom where the same patient variant has been associated with common variable immunodeficiency [33]. To our knowledge, this is one of the first reports describing the coexistence of this NLRP12 variant with autoinflammatory manifestations and antibody deficiency. However, given the current classification of the variant as benign and the absence of functional or segregation data in this case, our findings should be interpreted as a possible association rather than confirmation of pathogenicity.

Further large‐scale studies are required to better characterize the clinical variability of *NLRP12*‐associated phenotypes, clarify genotype–phenotype correlations, and explore potential therapeutic strategies targeting inflammasome pathways.

## Funding

No funding was received for this manuscript.

## Consent

All the patients allowed personal data processing and informed consent was obtained from all individual participants included in the study.

## Conflicts of Interest

The authors declare no conflicts of interest.

## Data Availability

The data that support the findings of this study are available from the corresponding author upon reasonable request.
